# Adenosine magnetic resonance imaging versus dobutamine stress echocardiography in patients with low probability for coronary artery disease

**DOI:** 10.1186/1532-429X-11-S1-O36

**Published:** 2009-01-28

**Authors:** Stamatios Lerakis, Athanasios V Anadiotis, Elisa Zaragoza-Macias, Emir Veledar, John Oshinski, Chris Vaccari, Akbar H Khan, Puneet Sharma, Irfan Shukrullah, Paolo Raggi, Arthur E Stillman

**Affiliations:** Emory University, Atlanta, GA USA

**Keywords:** Coronary Artery Disease, Ischemia, Aspirin, Baseline Characteristic, Chest Pain

## Introduction

Accurate assessment of patients with chest pain without electrocardiographic changes or elevation of serum cardiac enzymes is challenging. There is increased interest in the role of dobutamine stress echocardiography (DSE) and adenosine magnetic resonance imaging (AMRI) performed in the chest pain unit as a diagnostic method to rule out Coronary Artery Disease (CAD) as the cause of the chest pain in this population.

## Purpose

The purpose of this study was to compare DSE and AMRI in patients with low probability of CAD.

## Methods

Inclusion criteria for the study were patients with normal EKG (no signs of cardiac ischemia) and negative cardiac enzymes, who were admitted to the Cardiac Decision Unit (CDU) from 2006–2008 at Emory University Hospital. The diagnostic method used was chosen randomly by physician preference. T-test was used to assess differences in continuous variables, and X^2^ square to test differences in categorical variables between the two groups. Logistic regression was used to assess the likelihood of detecting CAD after adjusting for technique used and baseline characteristics.

## Results

A total of 306 patients were included, 103 patients were evaluated with AMRI and 203 underwent DSE. Mean age was similar among groups (52 for AMRI vs. 54 for DSE). Patients in AMRI group were more likely to be males, had more risk factors for CAD, and used more Beta blockers or aspirin at baseline compared to patients evaluated by DSE. AMRI identified more patients as having CAD compared to DSE (13 (12.6%) vs.3 (1.5%), p = < 0.0001). This difference remained significant even after adjusting for baseline characteristics and risk factors [OR of CAD by AMRI vs DSE = 7.01, CI (1.48–33.16) p = 0.014]. (Data in Table 1.)

**Table 1:** Table 1

Characteristics	MRI	DSE	p-value
AGE	52 ± 12	54 ± 13	0.0571
Gender (males)	144 (70.9%)	38 (38%)	<0.0001
CAD	16 (16%)	13 (6.4%)	0.0076
HTN	66 (64.7%)	111 (54.7%)	0.0941
DM	30 (29.7%)	36 (17.7%)	0.0171
SMOKING	20 (19.6%)	39 (19.5%)	0.2967
Dyslipidemia	40 (39.2%)	46 (22.8%)	0.0027
Family_history_of_CAD	52 (51%)	74 (54.4%)	0.6029
EF	65.0 ± 10.4	64.2 ± 6.1	0.4235
Coronoary Artery Disease	13 (12.6%)	3 (1.5%)	<0.0001
Beta Blocker use	29 (28.4%)	25 (12.4%)	0.0005
Ca_B	19 (18.6%)	27 (13.4%)	0.2268
ACEi	21 (20.6%)	21 (10.4%)	0.015
ARBs	14 (13.7%)	20 (9.9%)	0.3178
ASA	27 (26.5%)	21 (10.4%)	0.0003

## Conclusion

In this prospective study of patients with low probability of CAD, AMRI identified more cases of CAD than DSE even after adjusting for baseline characteristics. Although selection bias could account for part of these results, a higher sensitivity for AMRI is suggested.

